# Pathological Significance and Prognostic Roles of Indirect Bilirubin/Albumin Ratio in Hepatic Encephalopathy

**DOI:** 10.3389/fmed.2021.706407

**Published:** 2021-08-30

**Authors:** Yanling Li, Huiyuan Liu, Keng Chen, Xueheng Wu, Jiawen Wu, Zhenjun Yang, Leyi Yao, Guanmei Wen, Change Zhang, Xin Chen, Xiaohui Chen, Daolin Tang, Xuejun Wang, Jinbao Liu

**Affiliations:** ^1^The Second Affiliated Hospital of Guangzhou Medical University, Guangzhou, China; ^2^Guangzhou Municipal and Guangdong Provincial Key Lab of Protein Modification and Degradation Lab, State Key Lab of Respiratory Disease, School of Basic Medical Sciences, Guangzhou Medical University, Guangzhou, China; ^3^Guangzhou Eighth People's Hospital, Guangzhou Medical University, Guangzhou, China; ^4^Institute of Digestive Disease of Guangzhou Medical University, The Sixth Affiliated Hospital of Guangzhou Medical University, Qingyuan People's Hospital, Qingyuan, China; ^5^Department of Surgery, UT Southwestern Medical Center, Dallas, TX, United States; ^6^Division of Basic Biomedical Sciences, University of South Dakota Sanford School of Medicine, Vermillion, SD, United States

**Keywords:** hepatic encephalopathy, liver failure, indirect bilirubin, albumin, free bilirubin

## Abstract

**Background and Aim:** Hepatic encephalopathy (HE) is a neurological disease caused by severe liver disease. Early identification of the risk factor is beneficial to the prevention and treatment of HE. Free bilirubin has always been considered to be the culprit of neonatal kernicterus, but there is no research to explore its role in HE. In this study, we aim to study the clinical significance of the indirect bilirubin-albumin ratio in HE.

**Methods:** A retrospective case-control study of 204 patients with liver failure was conducted. Human serum albumin (HSA) or heme oxygenase-1 (HO-1) inhibitor SnPP (Tin protoporphyrin IX dichloride) was injected intraperitoneally into *Ugt1*^−/−^ mice to establish a treatment model for endogenous hyperbilirubinemia.

**Results:** IBil/albumin ratio (OR = 1.626, 95% CI1.323–2.000, *P* < 0.001), white blood cell (WBC) (OR = 1.128, 95% CI 1.009–1.262, *P* = 0.035), ammonia (OR = 1.010, 95% CI 1.001–1.019, *P* = 0.027), platelet (OR=1.008, 95% CI 1.001–1.016, *P* = 0.022), Hb (OR = 0.977, 95% CI 0.961–0.994, *P* = 0.007), and PTA (OR = 0.960, 95% CI 0.933–0.987, *P* = 0.005) were independent factors of HE. Patients with a history of liver cirrhosis and severe HE (OR = 12.323, 95% CI 3.278–47.076, *P* < 0.001) were more likely to die during hospitalization. HSA or SnPP treatment improved cerebellum development and reduced apoptosis of cerebellum cells.

**Conclusion:** The IBil/albumin ratio constitutes the most powerful risk factor in the occurrence of HE, and reducing free bilirubin may be a new strategy for HE treatment.

## Introduction

Hepatic encephalopathy (HE) is a syndrome caused by severe liver dysfunction, which is characterized by metabolic disorders and neurological dysfunction (1). The main hypotheses regarding the pathogenesis of HE include hyperammonemia, altered neurotransmission, and manganese toxicity (2). Among classical risk factors, hyperammonemia ranks top 1. However, most of the HE patients do not benefit from treatment targeting above-mentioned pathogenic processes (3), which indicates that other major causative factors are also involved.

Jaundice is one of the main clinical manifestations of severe liver diseases. Three pathological causes of elevated serum bilirubin in patients with severe liver disease are: intrahepatic and/or extrahepatic bile duct obstruction, hepatocyte degeneration and necrosis, and hemolysis due to hepatolenticular degeneration (4). The main clinical criteria for distinguishing them are the type of elevated bilirubin and the related jaundice. However, the potential unique effects of the physical and chemical properties of different types of bilirubin are unclear. Bilirubin is one of the products of heme metabolism during the destruction of senescent red blood cells (RBC). Then, the hemoglobin product is converted into carbon monoxide, iron, and biliverdin by heme oxygenase-1 (HO-1)-mediated oxidization. Biliverdin is converted to bilirubin by a reaction catalyzed *via* biliverdin-reductase. Bilirubin is then combined with albumin and transported to hepatocytes, where the endoplasmic reticulum uridine diphosphate glucuronosyl transferase 1 (UGT1) catalyzes the conjugation of bilirubin with glucuronic acid to form conjugated bilirubin (B_c_). Bilirubin that is not conjugated to glucuronic acid is called unconjugated bilirubin (B_u_), also known as indirect bilirubin (IBil) clinically. The part of B_u_ that is not bounded by albumin is referred to as free bilirubin (B_f_), which is fat-soluble and can penetrate cell membranes. Under normal circumstances, the content of B_f_ in the blood is very low. In liver failure, serum B_f_ may increase due to increased total bilirubin, insufficient albumin, and/or abnormal albumin binding capacity (5).

Previous studies have found that total bilirubin is related to kernicterus (6). However, with the increasing understanding and technology development of B_f_ detection, the role of B_f_ in neurological dysfunction has attracted great attention. At present, clinical and basic studies have revealed that B_f_, rather than total bilirubin in serum is an important cause of kernicterus in the newborn (7, 8). Lowering B_f_ can reduce the corresponding nerve damage in neonates. Moreover, Waser et al. reported a case of adult patient with severe HE suffered from kernicterus (9). However, whether B_f_ is involved in the occurrence and development of HE remains to be determined.

Increasing evidence shows that the albumin to bilirubin ratio (ALBI) grade is an important indicator for predicting the prognosis of patients with end-stage liver disease (including HE) (10, 11). In addition, albumin dialysis improves hepatic encephalopathy by decreasing serum bilirubin (12–14). None of these studies have revealed the relationship between B_f_ and HE.

To address the role of bilirubin in HE patients, a retrospective case-control study was first conducted to analyze the risk factors of liver failure patients for developing HE and in-hospital mortality. Next, we tested the effect of reducing free bilirubin by treatment with HSA or HO-1 inhibitor SnPP on cerebellum development and apoptosis in neonatal *Ugt1*^−/−^ mice, a genetic model of hyperbilirubinemia (15).

## Methods

### Ethics Approval and Consent to Participate

The protocol for the care and use of all animals in this study was in accordance with the Guangdong Animal Center for the ethical treatment of animals and approved by the Institutional Animal Care and Use Committee of Guangzhou Medical University (SYXK2016-0168, Guangzhou, China).

### Patients

To assess the risk factors of HE, we conducted a retrospective study in the intensive liver disease wards of the Eighth People's Hospital of Guangzhou. From January 2015 to December 2017,102 patients who were hospitalized and suffered from liver failure with HE were recruited consecutively. Through frequency matching by age and gender, another 102 patients with liver failure who were diagnosed with no HE were recruited. Diagnosis was confirmed by qualified physicians using standard criteria previously described (16–18). All patients were over 18 years old. The study excluded patients who were diagnosed or suspected of having liver cancer and women who were pregnant or breast feeding. Written informed consent was obtained from all participants, and the study was approved by the hospital ethical committee.

### Clinical Data

To determine the risk factors for the occurrence and severity of HE, blood samples were collected from each patient on admission. Blood tests were requested, including complete blood count (CBC), plasma bilirubin, albumin, ammonia, alanine aminotransferase (ALT), aspartate aminotransferase (AST), blood urea nitrogen (BUN), and prothrombin activity (PTA). The primary outcome was the occurrence of HE and the secondary outcome was the patient's mortality.

### Animals

The heterozygous *Ugt1* knockout (*Het;Ugt1*^+/−^) mice in the C57BL/6 background were a gift from the laboratory of Dr. Zhongqiu Liu of Guangdong University of Chinese Medicine (19). The homozygous *Ugt1* knockout mice (*Hom;Ugt1*^−/−^) were produced by breeding *Ugt1*^+/−^ mice at the Animal Center of Guangzhou Medical University. Het and wile-type (WT) littermates were used as controls. The mice were housed and handled according to institutional guidelines, and experimental procedures were approved by Guangzhou Medical University. Mice were kept in a temperature-controlled environment with a 12/12-hour light/dark cycle. They received a standard chow diet and water *ad libitum*. In this study, a total of 79 mice underwent three tests to evaluate the neurotoxicity of hyperbilirubinemia.

### General Appearance and Growth Pattern of Hom Mice

WT and Het littermates (*n* = 15) and Hom mice (*n* = 12) were raised with their mothers after birth. Body weight and survival status of each animal were monitored every day until death.

### Albumin Treatment

The *Ugt1*^−/−^ mouse is a lethal mouse model of neonatal hyperbilirubinemia, without the application of the standard phototherapy or albumin (20). Bilirubin does not cause lethality and obvious cerebellar abnormalities after P15 in mice (21). Considering all these above, in this study, Hom mice were administrated of HSA (AlbuRx,10 g/50 ml, CSL Behring) by intraperitoneal injection from postnatal day 1 (P1) to P7, with 5 g/kg/24 h (*n* = 13). On P8–P9, the mice were randomly divided into two groups. Five of them were received albumin treatment continuously, while the others were administrated of equivalent volume of phosphate buffered saline (PBS) instead. Using the same two-step treatment protocol, WT and Het littermates (*n* = 15) were used as controls.

### SnPP Treatment

Similarly, Hom mice were administrated of HSA (AlbuRx,10 g/50 ml, CSL Behring) by intraperitoneal injection from P1 to P7, with 5 g/kg/24 h (*n* = 9). On P8–P9, four of them were randomly received intraperitoneal injection of SnPP (Tocris Bioscience) with dose of 20 mg/kg on P8 and 30 mg/kg on P9. The dosage was modified from previous study (22). Meanwhile, others were administrated of equivalent volume of PBS instead. Using the same two-step treatment protocol, WT and Het littermates (*n* = 10) were used as controls.

### Biochemical Analysis of Serum Samples

Blood samples of HSA-treated mice were collected on P10 by cardiac puncture and stored in opaque Eppendorf tubes. The serum was extracted after centrifugation at 4 °C, 5000 rpm for 5 min. The serum samples were stored at −80 °C until analysis. ALT (L-type ALTIFCC, Wako, Japan), albumin(ALB II-HA, Wako, Japan), total bilirubin (Total BilE-HA, Wako, Japan), and direct bilirubin (Direct Bil E-HA, Wako, Japan) were assayed using an automatic analyzer (Hitachi Ltd. 3100 Serial, Tokyo, Japan).

### Cerebellar Histology

Cerebellum were removed from skulls. The cerebellum of each genotype was fixed with 4% paraformaldehyde (PFA) in PBS overnight at 4 °C. After cryoprotection in 30% sucrose, the cerebellum was divided into two halves along the median sagittal plane. Specimens were frozen in cryostat embedding medium, and first five 20 μm sagittal sections were obtained in a cryostat. Nissl staining was performed to assess the development of cerebellum in newborn mice according to the product protocol. The analysis of the layer thickness was performed by measuring the layer depth (μm) as previously described (21). For immunofluorescence, the sections were reheated for 30 min followed by 4% PFA fixation for 10 min at room temperature (RT). Then specimens were blocked for 1.5 h at RT with 5% bovine serum albumin (BSA) in PBS 0.1% Triton X-100. After blocking, the specimens were incubated with cleaved caspase3 primary antibody (1:1000,CST 9661) at 4°C overnight, and then incubated with donkey polyclonal secondary antibody to rabbit IgG-H&L (Alexa Fluor^®^ 555) (1:500, ab150074) for 1 h at RT. The nucleus was visualized by addition of DAPI (Abcam) for 3 min after secondary antibody solution. 3 × 5-min washes with PBST were performed between two steps. TUNEL assay was performed using the TUNEL Cytotoxicity Test Kit (KGA702, KeygenBiotech). The images of Nissl staining and TUNEL assay were acquired by the aperio digital pathology slide scanner (Leica, UK). Additionally, images of immunofluorescence were obtained by confocal microscopy (SP8, Leica).

### Statistical Analyses

Comparisons between two groups were performed by Mann–Whitney U test or *t* test for continuous variables and Chi-square test for categorical variables. One-way ANOVA followed by LSD tests was performed among group's comparisons. For univariate analyses, each variable was introduced into logistic regression analysis first. Then those factors showing a clinically and statistically significant (*p* < 0.05) association to the occurrence of HE were selected. The final models were fitted by STATA (version 14.0) conditional multivariate logistic regression in a step-wise forward method (Likelihood Ratio). For analyzing the risk factors of HE hospital death, STATA (version 14.0) multivariate logistic regression in a step-wise forward method was used. *P* < 0.05 was considered statistically significant.

## Results

### Characteristics of Liver Failure Patients With and Without HE

Patients with HE did not significantly differ from those without HE in relation to cirrhosis (64.71 vs. 60.78%) or the etiologies of liver failure (hepatitis B 88.24 vs.85.29%, hepatitis C/hepatitis V 4.90 vs. 1.96%, and the remaining causes 6.86 vs. 12.75%). Both groups of patients showed similar levels of hepatic failure and renal failure. However, the coagulation ability of HE patients seems to be lower [(PTA 22.57 (15.77, 33.06) % vs. 35.59 (27.13, 42.23) %, *P* < 0.001], but there was no difference in PLT [PLT 119.50 (73.00, 179.75) × 10^9^/L vs. 106.50 (78.75, 145.25) × 10^9^/L, *P* = 0.302].Among the classical causes of HE, patients with HE had higher serum ammonia levels [89.90 (55.08, 121.40) μmol/L vs. 49.70 (32.75, 72.05) μmol/L, *P* < 0.001]. Surprisingly, the degree of bacterial infection was higher in HE patients [WBC 8.55 (6.36, 12.95) × 10^9^/L vs. 6.73 (4.77, 9.17) × 10^9^/L, *P* < 0.001; proportion of neutrophils 79.35 (73.50, 84.68) % vs. 74.65 (64.58, 81.90) %, *P* = 0.006]. Serum bilirubin [TBil 415.48 (286.5, 492.03) μmol/L vs. 249.59 (164.84, 361.90) μmol/L, DBil 214.53 (154.60, 274.48) μmol/L vs. 142.71 (90.68, 210.28) μmol/L, IBil 184.34 (134.46, 233.04) μmol/L vs. 98.59 (66.83, 151.76) μmol/L, *P* < 0.001 respectively] was higher in HE patients. Accordingly, the serum bilirubin/albumin ratio was also higher in HE patients. As a result, the mortality of HE patients was twice that of non-HE patients [25.49% vs. 9.80%, *P* = 0.003] (Table 1).

**Table 1:** Comparisons of characteristics of patients with and without HE.

**Variables median** **(interquartile range)**	**Non-HE** **(*N* = 102)**	**HE** **(*N* = 102)**	***P* value**
Age, y	49 (39, 60.25)	49 (39, 61)	0.874
Male, *N* (%)	80 (78.43)	80 (78.43)	1.000
Cause, *N* (%)			0.250
Hepatitis B	87 (85.29)	90 (88.24)	
Hepatitis C/hepatitis V	2 (1.96)	5 (4.90)	
The other causes	13 (12.75)	7 (6.86)	
Cirrhosis, *N* (%)	62 (60.78)	66 (64.71)	0.562
WBC, × 10^9^/L	6.73 (4.77, 9.17)	8.55 (6.36, 12.95)	<0.001
Neutrophils, %	74.65 (64.58, 81.90)	79.35 (73.50, 84.68)	0.006
RBC, × 10^12^/L	3.90 (3.26, 4.30)	3.98 (3.11, 4.71)	0.725
Hb, g/L	117.00 (104.75, 133.00)	111.50 (89.75, 130.50)	0.034
PLT, × 10^9^/L	106.50 (78.75, 145.25)	119.50 (73.00, 179.75)	0.302
Ammonia, μmol/L	49.70 (32.75, 72.05)	89.90 (55.08, 121.40)	<0.001
TBil, μmol/L	249.59 (164.84, 361.90)	415.48 (286.5, 492.03)	<0.001
DBil, μmol/L	142.71 (90.68, 210.28)	214.53 (154.60, 274.48)	<0.001
IBil, μmol/L	98.59 (66.83, 151.76)	184.34 (134.46, 233.04)	<0.001
TBA, μmol/L	118.65 (87.09, 149.60)	132.54 (93.98, 162.73)	0.112
Albumin, g/L	28 (25, 32)	28 (26, 31)	0.897
ALT, U/L	412.00 (79.00, 955.50)	320.00 (103.75, 761.50)	0.697
AST U/L	294.50 (106.00, 706.25)	250.00 (138.75, 506.75)	0.602
BUN, mmol/L	4.18 (2.95, 6.35)	4.78 (3.04, 8.47)	0.133
Creatine, μmol/L	70.50 (59.00, 83.00)	74.00 (61.75, 101.25)	0.065
PTA, %	35.59 (27.13, 42.23)	22.57 (15.77, 33.06)	<0.001
TBil/Albumin ratio, μmol/g	8.97 (6.19, 13.04)	14.63 (10.64, 18.28)	<0.001
DBil/Albumin ratio, μmol/g	5.02 (3.53, 7.70)	7.55 (5.76, 9.94)	<0.001
IBil/albumin ratio, μmol/g	3.56 (2.46, 5.71)	6.80 (4.81, 8.25)	<0.001
Mortality, *N* (%)	10 (9.80)	26 (25.49)	0.003

*Categorical variables expressed as number (%), continuous variables as median (Q1, Q3). Mann-Whitney U test for continuous parameters, and Chi-squared test for categorical variables.WBC, white blood cells; RBC, red blood cells; Hb, hemoglobin; PLT, platelet; TBil, total bilirubin; DBil, direct bilirubin; IBil, indirect bilirubin; TBA, total bile acid; ALT, alanine aminotransferase; AST, aspartate aminotransferase; BUN, blood urea nitrogen; PTA, prothrombin activity*.

### Hospitalized Death of Liver Failure Patients With HE

During hospitalization, 26 patients with HE died. Compared with survivors with HE, non-survivors were older with a high proportion of cirrhosis and severer HE. There were no differences in the causes of liver failure, complete blood count, hepatic function, renal function, as well as the levels of serum bilirubin and bilirubin/albumin (Table 2).

**Table 2:** Comparison of characteristics of survivors and non-survivors during hospitalization with HE.

**Variables median** **(interquartile range)**	**Survivors** **(*N* = 76)**	**Non-survivors** **(*N* = 26)**	***P* value**
Age, y	47 (39, 58)	56 (41.75, 63.25)	0.043
Male, *N* (%)	60 (78.9)	20 (76.9)	0.828
Cause, *N* (%)			0.360
Hepatitis B	69 (90.8)	21 (80.8)	
Hepatitis C/hepatitis V	3 (3.9)	2 (7.7)	
The other causes	4 (5.3)	3 (11.5)	
Cirrhosis, *N* (%)	44 (57.89)	22 (84.62)	0.017
Grade of HE, *N* (%)			<0.001
I	13 (17.10)	0 (0)	
II	29 (38.16)	3 (11.54)	
III	20 (26.32)	9 (34.62)	
IV	14 (18.42)	14 (53.84)	
WBC, × 10^9^/L	8.52 (6.35, 13.24)	9.16 (6.39, 13.03)	0.701
Neutrophils, %	78.65 (73.50, 84.83)	81.05 (73.35, 84.68)	0.583
RBC, × 10^12^/L	4.13 (3.04, 4.68)	3.79 (3.18, 4.97)	0.939
Hb, g/L	111.50 (90.25, 133.00)	111.00 (89.25, 125.25)	0.939
PLT, × 10^9^/L	121.50 (73.00, 177.50)	109.50 (73.00, 191.00)	0.902
Ammonia, μmol/L	88.35 (56.58, 121.15)	93.75 (50.38, 125.33)	0.498
TBil, μmol/L	415.48 (312.84, 500.50)	415.07 (250.55, 470.38)	0.372
DBil, μmol/L	213.60 (159.14, 279.07)	222.29 (130.56, 260.45)	0.591
IBil, μmol/L	193.00 (136.19, 236.09)	160.81 (110.67, 229.15)	0.236
TBA, μmol/L	133.39 (95.19, 164.84)	130.15 (80.03, 160.21)	0.701
Albumin, g/L	28 (26, 32)	28.00 (24.50, 29.75)	0.518
ALT, U/L	370 (110, 873)	313 (86.50, 555.00)	0.388
AST U/L	234 (127, 497)	303 (146.50, 511)	0.426
BUN, mmol/L	4.78 (2.69,8.64)	4.86 (3.45,8.47)	0.872
Creatine, μmol/L	73.50 (61.25, 105.75)	77.00 (61.00, 98.75)	0.724
PTA, %	22.25 (15.07, 33.18)	24.03 (16.05, 30.21)	0.631
TBil/Albumin ratio, μmol/g	14.34 (10.70, 18.41)	15.15 (9.24, 16.93)	0.426
DBil/Albumin ratio, μmol/g	7.48 (5.89, 9.94)	8.08 (5.48, 9.97)	0.903
IBil/albumin ratio, μmol/g	6.90 (4.98, 8.54)	6.47 (4.44, 8.05)	0.242

*Categorical variables expressed as number (%), continuous variables as median (Q1, Q3). Mann-Whitney U test for continuous parameters, and Chi-squared test for categorical variables.WBC, white blood cells; RBC, red blood cells; Hb, hemoglobin; PLT, platelet; TBil, total bilirubin; DBil, direct bilirubin; IBil, indirect bilirubin; TBA, total bile acid; ALT, alanine aminotransferase; AST, aspartate aminotransferase; BUN, blood urea nitrogen; PTA, prothrombin activity*.

### Risk Factors of HE and Hospitalization Death of HE Patients

The STATA conditional univariate logistic regression analysis was used to evaluate the possible risk factors of HE. Within the twelve factors related to HE development, using a step-wise forward method, IBil/albumin ratio, WBC, ammonia, PLT, Hb, and PTA were proved to be independent related to HE (Table 3).

**Table 3:** Independent predicting factors for HE.

**Variables**	**β**	**OR**	**95% CI**	***P* value**
IBil/albumin ratio	0.486	1.626	1.323–2.000	<0.001
WBC	0.121	1.128	1.009–1.262	0.035
Ammonia	0.010	1.010	1.001–1.019	0.027
PLT	0.009	1.008	1.001–1.016	0.022
PTA	−0.041	0.960	0.933–0.987	0.005
Hb	−0.023	0.977	0.961–0.994	0.007

*WBC, white blood cells; PLT, platelet; PTA, prothrombin activity; Hb, hemoglobin*.

For the risk factors of hospitalization death in HE patients, none of the blood results were related to this problem. However, patients with a history of liver cirrhosis (OR = 5.895, 95% CI 1.708–12.422, *P* = 0.005) and severe HE (OR = 12.323, 95% CI 3.278–47.076, *P* < 0.001) were more likely to die during hospitalization.

### Loss of *Ugt1* Induces Apoptosis in Cerebellum Cells

**Figure 1:**
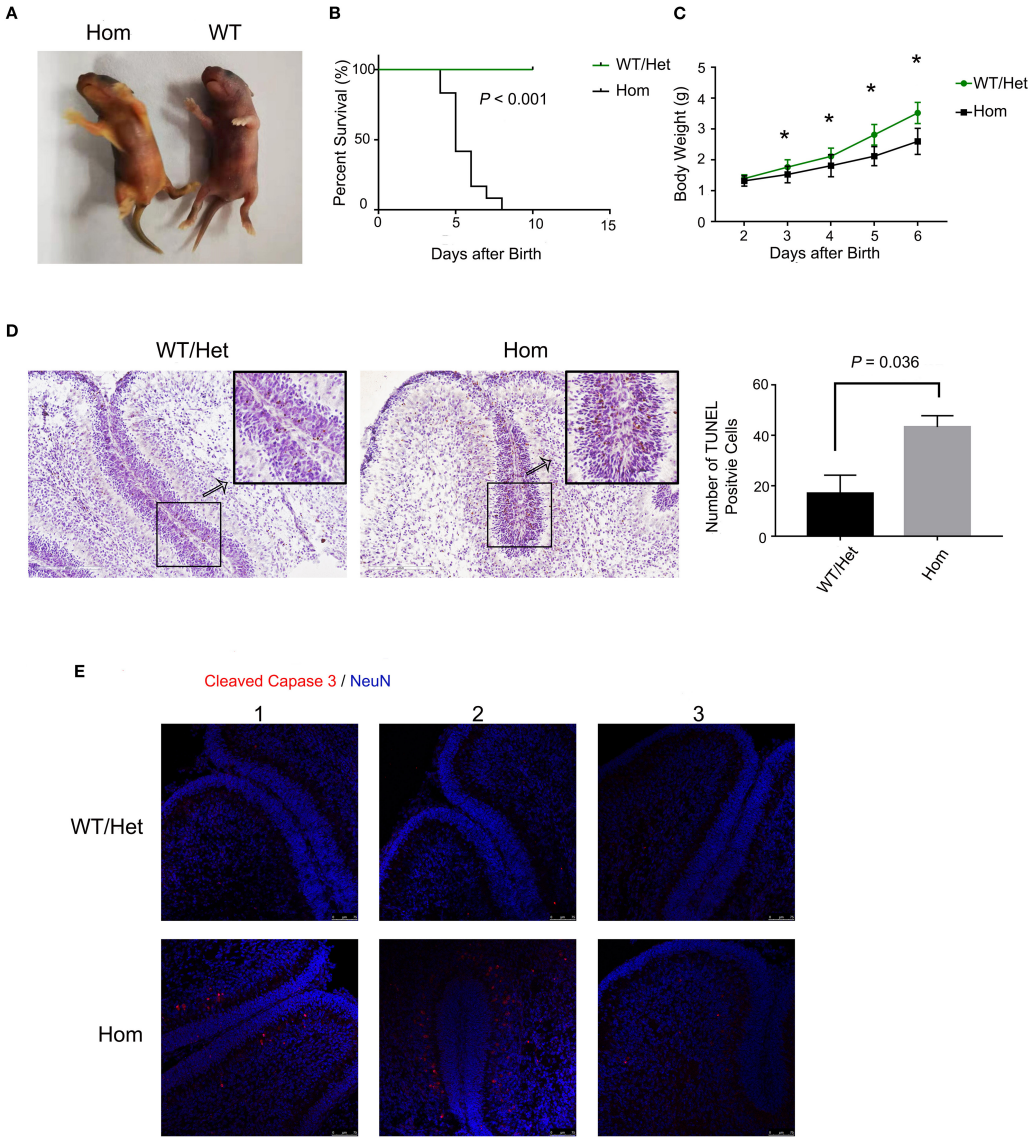
Ugt1 mutation induces apoptosis of cerebellum cells. **(A)** Appearance of jaundiced *Ugt1*^−/−^mice at P4. **(B)** Kaplan-Meier survival curves of Ugt1WT/Het mice (*n* = 14) and Hom mice (*n* = 12). **(C)** Comparison of body weight of Ugt1WT/Het mice (*n* = 14) and Hom mice (*n* = 12). Since all Hom mice died within 7 days after birth without any treatment, the statistic was done from P2 to P6 using *t* test. *represented *P* value of comparing body weight between the two groups on the same day after deliver (*P* < 0.05). **(D)** Left panel, representative TUNEL images on the cerebellar frozen section at P4. Scale bar = 200 μm. Right panel, TUNEL positive cells were counted from lobule 4 to lobule 6, *t* test was performed (*n* = 3, respectively). **(E)** Representative fluorescent immunohistochemistry of cerebellar frozen sections with an anti-cleaved caspase 3 antibody (red) from WT/Het and *Ugt1*^−/−^ mice at P4 (*n* = 3, respectively). DAPI (blue) was used to mark nuclei. Scale bar = 75 μm.

Hyperbilirubinemia is the main manifestations of patients with liver failure. It is worth studying the neurotoxicity of hyperbilirubinemia and the clinical benefit of lowering serum bilirubin. In animal experiments, we applied *Ugt1*^−/−^ mice, the well-known lethal model of hyperbilirubinemia. The deficiency of UGT1, the catalyzing enzyme for the conjugation of bilirubin, in mice can mimic liver failure patients with hyperbilirubinemia. *Ugt1*^−/−^ mice showed the appearance of jaundice even within a few hours after delivery, which was the major distinguishing feature from their WT and Het littermates (Figure 1A). In addition, due to P3, the weight of *Ugt1*^−/−^ mice was significantly lower than that of WT and Het littermates, and was about 25% lower than that of P5 (Figure 1C). As a result, without any treatment, more than 50% of the *Ugt1*^−/−^ mice died on P5, and the remaining mice could not survive beyond P7 (Figure 1B). The cerebellum is proved to be a target of toxic substances, including bilirubin, ethanol, and diphenylhydantoin. The cerebellum is highly vulnerable during neurodevelopment (23, 24). Vodret et al. reported that bilirubin not only induced apoptosis in the cerebellum of *Ugt1*^−/−^ mice under the C57BL/6 background, but also under the FVB/NJ background (25, 26). To confirm the neurotoxicity of bilirubin toward the cerebellum, we performed TUNEL assay (Figure 1D) and immunofluorescence staining of cleaved caspase3 (Figure 1E), which is the key executioner of apoptosis. We found a significant increase in TUNEL and cleaved caspase3 positive cells in the cerebellum of *Ugt1*^−/−^ mice (*P* < 0.05).

### HSA Treatment Improves Cerebellum Development and Reduces Apoptosis of Cerebellum Cells in *Ugt1*^–/–^ Mice

**Figure 2:**
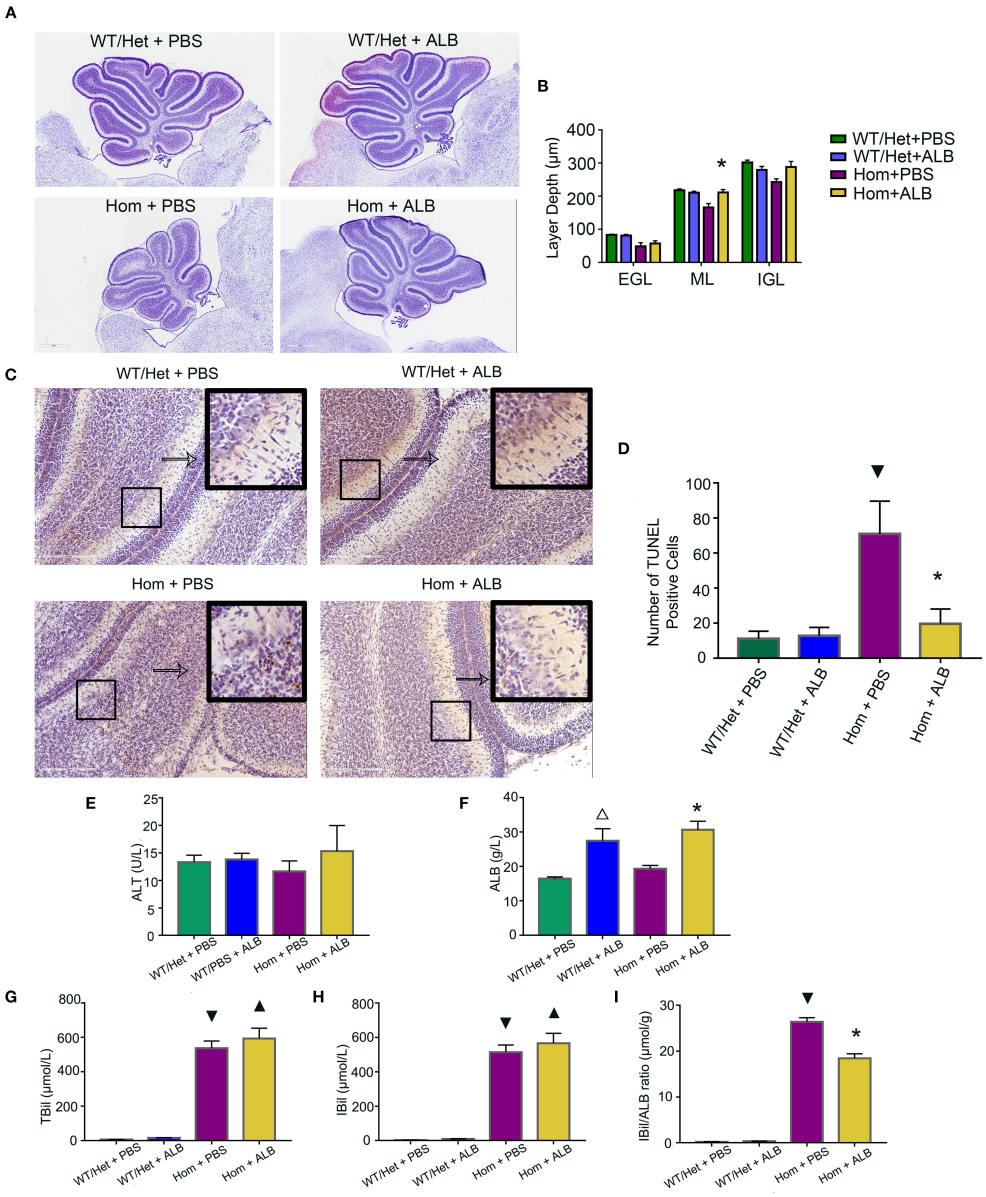
HSA treatment improves cerebellum development and reduces apoptosis of cerebellum cells. **(A)** Representative images of Nissl staining of the cerebellum of Ugt1 WT/Het and Hom mice that received continuous HSA or withdrawn from the HSA on P8–P9. Scale bar = 600 μm. **(B)** One-way ANOVA analysis was conducted among four groups of mice at every layer depth of lobule 4 and lobule 9 (*n* = 5–6, respectively). Compared with Hom + PBS mice, HSA treatment for another two days increased the ML depth of the cerebellum of *Ugt1*^−/−^ mice. **(C,D)** Left panel, representative TUNEL images on the cerebellar frozen section at P10. Scale bar = 200 μm. Right panel, TUNEL positive cells were counted (*n* = 3–4, respectively). **(E–I)** Blood tests of hepatic function (*n* = 5–8, respectively). Serum ALT levels did not show difference among groups. However, the serum bilirubin level was higher in the *Ugt1*^−/−^group. Two more days of HSA treatment increased serum ALB levels in WT/Het + ALB group or Hom + ALB group. The IBil/ALB ratio was significantly lower in the Hom + ALB group than in the Hom + PBS group. ^▾^represented *P* value comparing between Hom + PBS group and WT/Het + PBS group (*P* < 0.05). *represented *P* value comparing between Hom+ALB group and Hom+PBS group (*P* < 0.05). ^Δ^represented *P* value comparing between WT/Het+ALB group and WT/Het+PBS group (*P* < 0.05). ^▴^represented *P* value comparing between Hom + ALB group and WT/Het + ALB group (*P* < 0.05). EGL, external granular layer; ML, molecular layer; IGL, internal granularlayer; ALT, alanine aminotransferase; ALB, albumin; TBil, total bilirubin; IBil, indirect bilirubin; IBil/ALB ratio, indirect bilirubin/albumin ratio.

B_f_, but not total bilirubin, is the prime culprit of neonatal kernicterus (27). Moreover, serum albumin as a binding ligand for B_f_ represents a novel treatment for neonatal kernicterus (28). Can reduction of B_f_ by supplementing albumin reduce the apoptosis of cerebellum cells in mice with hyperbilirubinemia? To answer this important question, all experimental animals received HSA for the first 7 days (5 g/kg, every 24 h), but starting from P8, each genotype group was randomly divided into two subgroups: one continuing to receive the HSA treatment for two additional days (Hom + ALB, WT/Het + ALB), while the other's treatment being switched to an equal volume of PBS (Hom + PBS, WT/Het + PBS). Nissl assay was conducted to evaluate cerebellum development by measuring the depth of lobule 4 and 9 of each layer. The cerebellum of Hom mice was smaller than that of WT/Het mice. HSA treatment for two extra days had a moderate but statistically significant effect on molecular layer (ML) depth in Hom mice; the ML layer depth of the Hom+ALB group was statistically significantly greater than that of the Hom+PBS group (*P* < 0.05; Figures 2A,B). The results of TUNEL assay revealed that the Hom + ALB mice had fewer TUNEL positive cells than Hom+PBS group (*P* < 0.05; Figures 2C,D). The blood test confirmed that the serum bilirubin levels were drastically higher in *Ugt1*^−/−^ mice compared with WT/Het littermates (Figures 2G,H). Two more days after HSA treatment, the serum albumin levels in both WT/Het + ALB and Hom+ALB groups were higher than in the PBS control treatment group (Figure 2F). Since ALB may restrict bilirubin from crossing the blood-brain barrier (BBB), the indirect bilirubin to albumin ratio (IBil/ALB ratio), which may reflect the level of B_f_, was significantly lower in the Hom+ALB group than that of the Hom + PBS group (Figure 2I).

### SnPP Treatment Improves Cerebellum Development and Reduces Apoptosis of Cerebellum Cells in *Ugt1^−/−^* Mice

**Figure 3:**
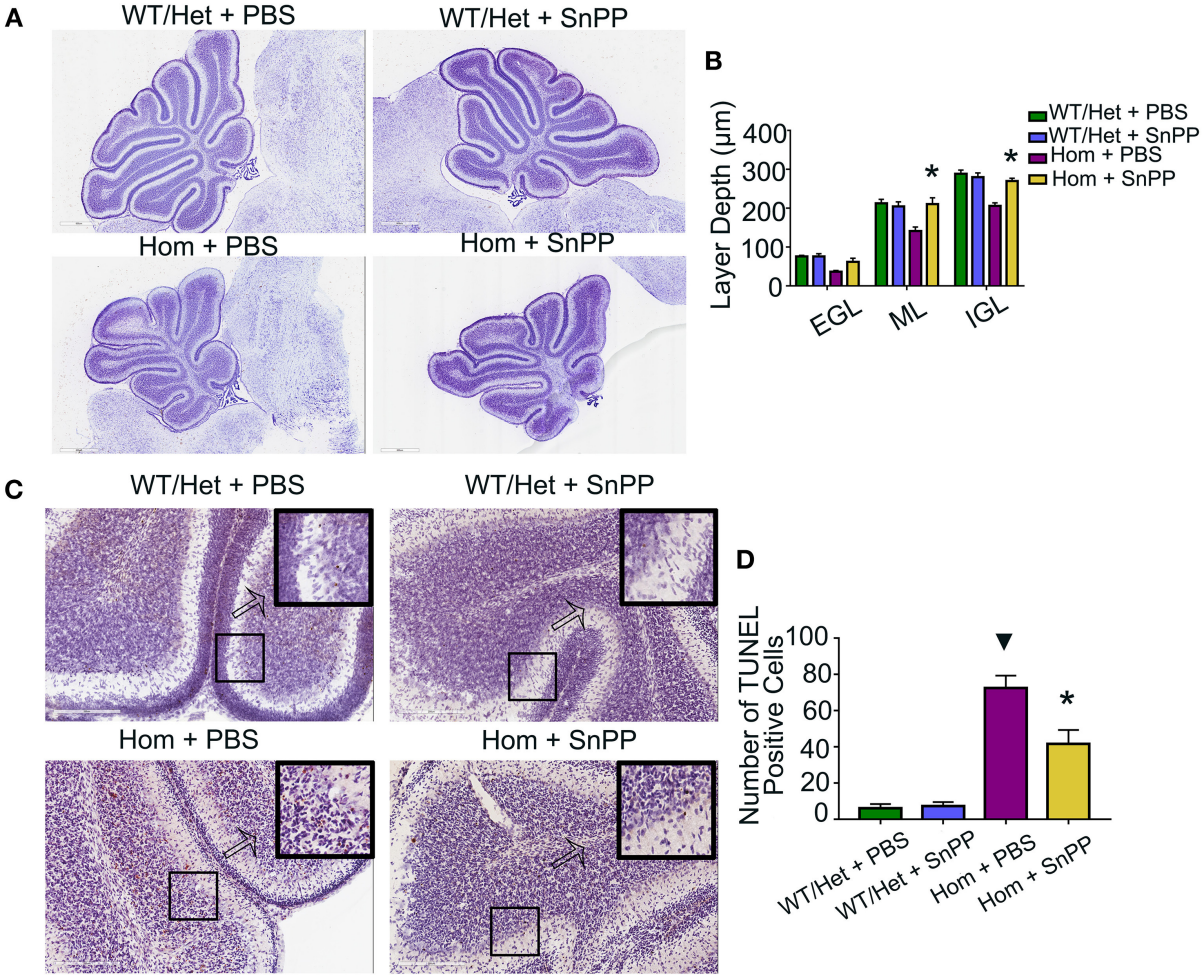
SnPP treatment improves cerebellum development and reduces apoptosis of cerebellum cells. **(A)** Representative images of Nissl staining of Ugt1 WT/Het and Hom mice cerebellum which were received SnPP (20 mg/kg P8 and 30 mg/kg P9) or equivalent volume of PBS. Scale bar = 600 μm. **(B)** One-way ANOVA analysis was conducted among four groups of mice at every layer depth of lobule 4 and lobule 9 (*n* = 4–6, respectively). Two days of SnPP treatment increased *Ugt1*^−/−^mice the ML depth and IGL depth of cerebellum when compared with Hom + PBS mice. **(C,D)** Left panel, representative TUNEL images on the cerebellum frozen section at P10. Scale bar = 200 μm. Right panel, TUNEL positive cells were counted (*n* = 3–4, respectively). ^▾^represented *P* value comparing between Hom+PBS group and WT/Het + PBS group (*P* < 0.05), *represented *P* value comparing between Hom + SnPP group and Hom+PBS group (*P* < 0.05). EGL, external granular layer; ML, molecular layer; IGL, internal granular layer.

HO-1 is a rate-limiting enzyme used to produce bilirubin. The HO-1 inhibitor SnPP can block HO-1, resulting in a reduction of bilirubin (29). To test the hypothesis that bilirubin leads to cerebellar dysplasia and apoptosis, we treated the Hom and WT/Het littermate mice with daily intraperitoneal injections of HSA (5 g/kg) for seven days (P1 through P7). On P8, each group was randomly divided into two sub-groups: one received SnPP treatment (20 mg/kg at P8 and 30 mg/kg at P9, i.p.), and the other group was treated with an equal volume of PBS. Similar to HSA treatment, SnPP treatment prevented the cerebellum development from retardation in *Ugt1*^−/−^ mice. The SnPP treatment did not affect the cerebellum development of WT/Het mice. The depth of ML and internal granular layer (IGL) of Hom+PBS mice was remarkably smaller than that of WT/Het+PBS mice, but this deficit was not found in the Hom + SnPP group. The depth of ML and IGL of Hom + SnPP mice was greater than that of the Hom+PBS group (*P* < 0.05, respectively) (Figures 3A,B). The results of TUNEL assay showed that the Hom + SnPP mice had fewer TUNEL positive cells than the Hom+PBS group (*P* < 0.05) (Figures 3C,D).

## Discussion

HE is a prevalent and life-threatening complication of both chronic liver disease and acute hepatic failure. Currently, clinical treatment of HE is mainly aimed at ammonia, a core player in the pathogenesis of HE. However, other factors, such as systemic and neuroinflammation, cell senescence, as well as derangements in blood-brain barrier (BBB) permeability/function, cerebrospinal fluid composition, glymphatic flow, cerebral energy metabolism, neurotransmission, and cell-cell communication, are also implicated in the neurological impairment of HE and provide potentially additional therapeutic targets (30). Here, our clinical case-control study reveals for the first time that the IBil/albumin ratio is the most powerful risk factor for HE in patients with liver failure, suggesting that free bilirubin may be a pathogenic factor for the neurological impairment of HE. Our experiments using a genetic model of hyperbilirubinemia further demonstrate that elevated levels of indirect bilirubin can cause neural cell death and retards neurodevelopment. Importantly, these changes can be remarkably attenuated by the administration of albumin or by pharmacological inhibition of bilirubin production. Hence, our current study not only identified free bilirubin as a potential pathogenic factor for HE, but also provided experimental evidence that this pathogenic factor can be effectively targeted to ameliorate neural damage.

In the case-control study performed here, we found that IBil/albumin ratio, WBC, ammonia, and PLT are independent risk factors for HE, while PTA and Hb are protective factors. Among them, the odd ratio of IBil/albumin ratio is the greatest, indicating that it is the most valuable predictive factor. Indirect bilirubin, also known as unconjugated bilirubin, binds to albumin in the blood during its transport to liver cells, where it is converted into conjugated bilirubin *via* glucuronidation that is catalyzed by UGT1. Hence, the conversion from unconjugated bilirubin to conjugated bilirubin in hepatocytes could be reduced, leading to the accumulation of unconjugated bilirubin in the circulation and an increase in the IBil/albumin ratio. When liver cells are more severely damaged and liver function is more impaired (usually seen in HE-related diseases), they may in turn increase free bilirubin even if the albumin level does not decrease. In addition to hyperbilirubinemia, liver failure, even cirrhosis could lead to low affinity between albumin with bilirubin (31). The increased lipid-soluble free bilirubin can pass through the BBB (20) and easily enter the brain, leading to brain dysfunction, Increased BBB permeability or BBB dysfunction associated with the onset of HE can further exacerbate this situation (32, 33).

Other risk factors we demonstrated are no different from previous studies. Lower hemoglobin and PTA levels have been shown to be associated with HE or liver failure (34, 35). Systemic inflammation is common in liver failure. This process involves in microglial activation, increasing levels of proinflammatory cytokines and neuroinflammation, and finally leading to encephalopathy (36). These findings were complementing to what we found in this study that WBC was the independent risk factor of HE. Despite conflicting results in the literature, hyperammonemia is considered to be the core pathogenesis of HE (37). Although we have similar findings that ammonia may play an important role in the occurrence of HE, the OR value is not the most powerful. Liver failure is often accompanied by complex alternations in the hemostatic system. Recent evidence shows that platelet activation in patients with liver cirrhosis is increased, and platelet counts in animal models of liver fibrosis are increased, which may be related to inflammation (38, 39). Overall, the risk factors we reported in this manuscript are reasonable.

B_f_ is neurotoxic and has been considered as a causative agent for neonatal kernicterus. Both neurons and glial cells are vulnerable to B_f_ (40). It has proved that B_f_ can participate in a new pathway of neurotoxicity by disrupting the calcium homeostasis in neonatal hippocampal neurons (41). We have previously shown that bilirubin precedes apoptosis by inhibiting the proteolytic function of proteasome (42), providing another mechanism for bilirubin neurotoxicity. The massive apoptosis was observed in the brains of *Ugt1* deficient mice (Figure 1). Cell death was blocked by the supplementation of albumin (Figure 2) or by treatment with SnPP (Figure 3), further confirming the neurotoxicity of free bilirubin. Indeed, persistent brain malfunction has been observed in patients recovering from HE (43, 44), consistent with permanent brain damage caused by neural cell death. Thus, we believed that increased B_f_ contributes to HE pathogenesis. Since we were unable to measure B_f_ in this study, we used the IBil/albumin ratio instead for the analysis. At admission, the higher the IBil/albumin ratio, the higher possibility of HE; hence, the IBil/albumin ratio also represents a good biomarker or predictor for HE. This finding is supported by previous studies. For example, Guevara et al. found that bilirubin exceeding 1.9 mg/dL (HR = 1.87, 95% CI 1.35–2.59) is a risk factor for dominant HE (45). Tapper et al. demonstrated that high bilirubin (HR = 1.07, 95% CI 1.05–1.09) is a risk factor for HE in patients with liver cirrhosis, and high albumin (HR = 0.54, 95% CI 0.48–0.59) is a protective factor (46). Chinese scholars revealed that resting MRI showed abnormal brain function in patients with liver cirrhosis and normal blood ammonia, but indirect bilirubin was elevated (47). These studies are also consistent with our claim that B_f_ contributes to brain dysfunction in patients with severe liver disease.

The cerebellum is vulnerable in hyperbilirubinemia (48, 49). HE is also associated with regional differential alterations. The cerebellum is one of the regions that of high susceptibility in HE, which has been proved in patient imaging examinations and various animal experiments (50–52). Apoptosis is one of the major ways leading to neurologic damage (53). Thus, in this study, apoptosis of cerebellum cells was investigated. Although the study on the mechanism of bilirubin-induced cerebellar neurotoxicity is based on neonatal jaundice, increased cerebellar blood flow in HE patients (54) may support that hyperbilirubinemia causes similar damage in severe liver disease. We used two treatment methods to evaluate whether attempts to lower B_f_ will produce good results. Indeed, not only HSA treatment, but also SnPP treatment, can improve apoptosis of cerebellum cells and maintain normal morphology, which can be achieved by reducing the IBil/albumin ratio. This is the same case that occurs in HE. SnPP as an inhibitor of HO-1 may decrease the antioxidant effect of HO-1. Nevertheless, the product of HO-1 oxidation could generate secondary inflammatory response, leading to brain impairment (55). For investigating the most appropriate application of SnPP or other HO-1 inhibitors, further studies are needed.

In summary, patients with liver failure with a higher IBil/albumin ratio are prone to HE. The congenital hyperbilirubinemia model of *Ugt1*^−/−^ mice with an abnormal cerebellum development and apoptosis can be rescued by treatment with albumin or HO-1 inhibitor (SnPP). Controlling B_f_ can treat neonatal bilirubin encephalopathy, and whether controlling B_f_ level can treat HE remains to be further studied.

## Data Availability Statement

The raw data supporting the conclusions of this article will be made available by the authors, without undue reservation.

## Ethics Statement

The studies involving human participants were reviewed and approved by The ethical committee of the second affiliated hospital of guangzhou medical university. The patients/participants provided their written informed consent to participate in this study. The animal study was reviewed and approved by Institutional Animal Care and Use Committee of Guangzhou Medical University (SYXK2016-0168, Guangzhou, China). Written informed consent was obtained from the individual(s) for the publication of any potentially identifiable images or data included in this article.

## Author Contributions

JL designed research studies, HL and KC collected the clinical data, YL performed most of the experiments, XWu, JW, ZY, and LY assisted in some of the experiments. YL analyzed data and wrote the manuscript. GW, CZ, and XiaC helped in data interpretation. DT, XinC, XWa, and JL revised the manuscript. All authors read and approved the final manuscript.

## Conflict of Interest

The authors declare that the research was conducted in the absence of any commercial or financial relationships that could be construed as a potential conflict of interest.

## Publisher's Note

All claims expressed in this article are solely those of the authors and do not necessarily represent those of their affiliated organizations, or those of the publisher, the editors and the reviewers. Any product that may be evaluated in this article, or claim that may be made by its manufacturer, is not guaranteed or endorsed by the publisher.
